# Decoy Exosomes Offer Protection Against Chemotherapy‐Induced Toxicity

**DOI:** 10.1002/advs.202203505

**Published:** 2022-09-04

**Authors:** Miao Fan, Hang Li, Deliang Shen, Zhaoshuo Wang, Huifang Liu, Dashuai Zhu, Zhenzhen Wang, Lanya Li, Kristen D. Popowski, Caiwen Ou, Kaihan Zhang, Jinchao Zhang, Ke Cheng, Zhenhua Li

**Affiliations:** ^1^ Affiliated Dongguan Hospital Southern Medical University Dongguan 523059 China; ^2^ College of Chemistry & Environmental Science Chemical Biology Key Laboratory of Hebei Province Key Laboratory of Medicinal Chemistry and Molecular Diagnosis of the Ministry of Education Hebei University Baoding 071002 China; ^3^ Department of Cardiology The First Affiliated Hospital of Zhengzhou University Zhengzhou Henan 450052 China; ^4^ College of Pharmaceutical Science Key Laboratory of Pharmaceutical Quality Control of Hebei Province Hebei University Baoding 071002 China; ^5^ Department of Molecular Biomedical Sciences and Comparative Medicine Institute North Carolina State University Raleigh NC 27607 USA; ^6^ Joint Department of Biomedical Engineering University of North Carolina at Chapel Hill and North Carolina State University Raleigh NC 27695 USA; ^7^ Guangdong Provincial Key Laboratory of Cardiac Function and Microcirculation Guangdong 510515 China; ^8^ Department of Chemistry The University of Manchester Manchester M13 9PL UK

**Keywords:** cardio‐oncology, cardiotoxicity, chemotherapy, exosome, tetrahedral DNA nanostructure

## Abstract

Cancer patients often face severe organ toxicity caused by chemotherapy. Among these, chemotherapy‐induced hepatotoxicity and cardiotoxicity are the main causes of death of cancer patients. Chemotherapy‐induced cardiotoxicity even creates a new discipline termed “cardio‐oncology”. Therefore, relieving toxicities induced by chemotherapy has become a key issue for improving the survival and quality of life in cancer patients. In this work, mesenchymal stem cell exosomes with the “G‐C” abundant tetrahedral DNA nanostructure (TDN) are modified to form a decoy exosome (Exo‐TDN). Exo‐TDN reduces DOX‐induced hepatotoxicity as the “G‐C” base pairs scavenge DOX. Furthermore, Exo‐TDN with cardiomyopathic peptide (Exo‐TDN‐PCM) is engineered for specific targeting to cardiomyocytes. Injection of Exo‐TDN‐PCM significantly reduces DOX‐induced cardiotoxicity. Interestingly, Exo‐TDN‐PCM can also promote macrophage polarization into the M2 type for tissue repair. In addition, those decoy exosomes do not affect the anticancer effects of DOX. This decoy exosome strategy serves as a promising therapy to reduce chemo‐induced toxicity.

## Graphical abstract

Chemotherapy is effective but can induce severe liver damage and cardiotoxicity. A new discipline called “cardio‐oncology” is developed rapidly to address those concerns. In this work, exosomes are used as a carrier for tetrahedral DNA nanostructure (TDN) to form a decoy exosome (Exo‐TDN) that can absorb excess DOX and mitigate cardiac and liver toxicity caused by DOX therapy.

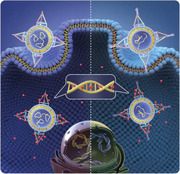

## 1 Introduction

Chemotherapy is still one of the most commonly used cancer therapies providing beneficial outcomes to patients. The remarkable progress of chemotherapy has enabled the prolonged survival time of cancer patients, which has led to the definition of cancer as a chronic disease.^[^ ^1^ ^]^ However, due to the nonspecific distribution of chemotherapeutic drugs, the organ toxicity of anticancer drugs has become a primary cause of treatment‐related deaths of cancer survivors.^[^ ^2^ ^]^ Liver plays a central role in drug metabolism and clearance. Studies have shown that >60% of chemotherapeutic drugs concentrate in the liver area, causing severe liver toxicity. Drug‐induced liver injury (DILI) is the main cause of acute liver failure, and is also a common cause of the termination of clinical trials.^[^ ^3^ ^]^ Researchers have designed nanoparticle drug delivery systems to increase the accumulation of chemotherapeutic drugs in tumor tissues to reduce their side effects. Half of such nano‐drugs, however, are still cleared by the reticuloendothelial system through the circulation, and thus accumulate in liver and cause serious hepatotoxicity.^[^ ^4^ ^]^ In addition, many chemotherapeutic drugs cause specific organ damage. For example, doxorubicin (DOX) is a chemotherapeutic drug that has strong cardiotoxicity.^[^ ^5^ ^]^ Studies have shown that >400 mg m^−2^ dose of DOX would induce heart failure.^[^ ^6^ ^]^ In addition to DOX, other anticancer drugs such as alkylating agents (cyclophosphamide, cisplatin, etc.) and antimetabolic drugs (fluorouracil) are also widely recognized to induce a strong cardiotoxicity. Moreover, owing to the extensive attention to chemotherapy‐induced cardiotoxicity, a new discipline called “cardio‐oncology” has emerged since the late 1990s owing to the high morbidity and mortality in the oncologic population. As such, reducing the toxicity of major organs throughout the treatment of cancer patients has become a key issue for improving the survival and quality of life of cancer patients.

**Scheme 1:**
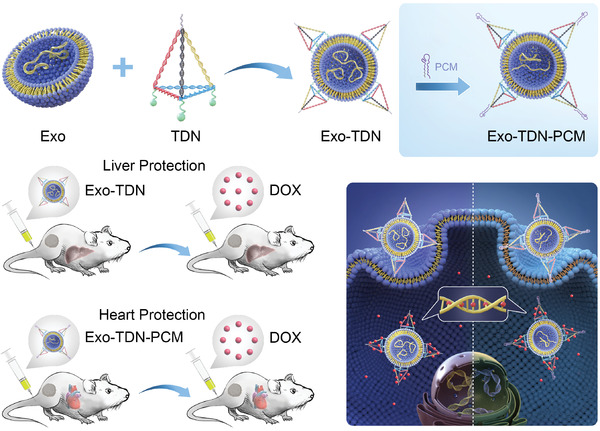
Schematic showing the synthesis of tetrahedral DNA with/without myocardial targeted peptides engineered exosomes (Exo‐TDN/Exo‐TDN‐PCM) and their detoxifying ability to DOX‐induced hepatotoxicity and cardiotoxicity.

Exosomes are sac‐like vesicles secreted by living cells that contain the same genetic material as parental cells, such as proteins, DNAs, and RNAs.^[^ ^7^, ^8^, ^9^, ^10^ ^]^ Studies have shown that exosomes derived from stem cells have the same repairing functions for damaged tissue.^[^ ^11^, ^12^, ^13^ ^]^ By analyzing the composition of exosomes, studies have found that a variety of miRNA strands in exosomes, such as miR‐223, miR‐21, and miR‐210, can inhibit apoptosis.^[^ ^14^, ^15^, ^16^ ^]^ Furthermore, stem cell derived exosomes have been reported to inhibit inflammation by polarizing M1‐type macrophages into the M2 type.^[^ ^17^, ^18^ ^]^ In this work, we used mesenchymal stem cell exosomes as a repairing carrier in combination with the “G‐C” abundant DNA nanostructure to synthesize a “detoxification repair agent”. GC‐rich DNA nanostructure has been widely used for DOX delivery systems owing to its high‐binding efficiency. Particularly, tetrahedral DNA with more G‐C sequences is more stable than bare double‐stranded DNA (dsDNA).^[^ ^3^, ^19^ ^]^ Therefore, our Exo‐TDN enters liver tissue through metabolic routes, thereby relieving DOX‐induced liver toxicity. In addition to protecting the liver from damage, the liver detoxicant agent would also promote the repair of a damaged liver owing to the presence of exosome carriers.^[^ ^20^, ^21^ ^]^ Importantly, the detoxification repair agent has been used to relieve cardiotoxicity by engineering myocardial targeted peptides on TDN. Exosomes from mesenchymal stem cells have also been shown to play an anti‐inflammatory role by inducing macrophage polarization, thereby promoting healing of damaged heart tissue.^[^ ^22^, ^23^ ^]^ In addition, by changing the target peptide, the system can be promoted to reduce the toxicity to multiple organs caused by DOX. The development of detoxification repair agents reduce the organ toxicity caused by chemotherapy and improve the survival time of cancer patients, which thus gives them important research significance (**Scheme** **1**).

## 2 Results

### 2.1 The Construction of Exo‐TDN

**Figure 1:**
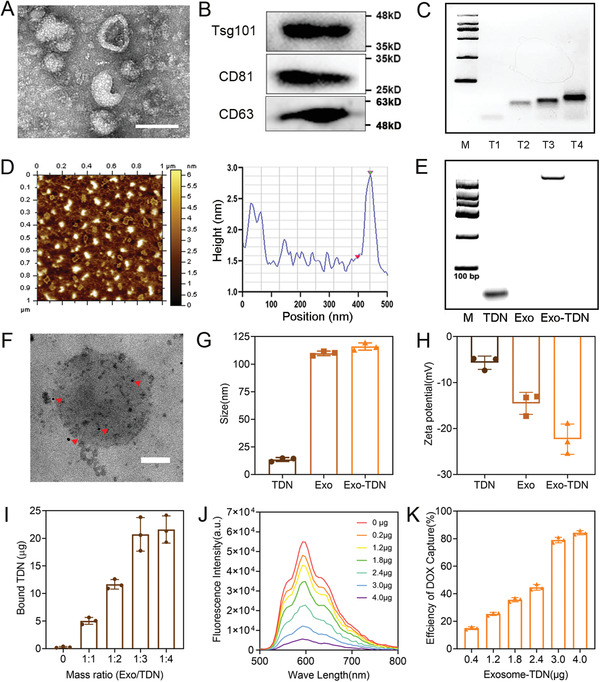
Characterization of Exo‐TDN. A) TEM picture of exosomes. Scale bar, 100 nm. B) Western blot analysis of exosomes. CD81, CD63, and TSG101 were exosome markers. C) Agarose electrophoresis of TDN (T1: S1; T2: S1+S2; T3: T2+S3; T4: T3+S4). D) AFM image of TDN and height distribution histograms. E) Agarose electrophoresis analysis of the synthesis of Exo‐TDN. F) TEM image of Exo‐TDN with immunogold labeling of TDN. Scale bar, 100 nm. G,H) NTA (G) and Zeta potential (H) analysis of TDN, Exo, and Exo‐TDN. I) The grafting efficiency of TDN on the exosome (*n* = 3). J) The fluorescence sp of 1 µmol L^−1^ DOX solution after adding different amounts of Exo‐TDN. K) The capture efficiency of DOX by Exo‐TDN (*n* = 3). Data are presented as the mean ± SD. *p <  0.05; ^**^ *p* < 0.01; ^***^ *p* < 0.001; ^****^ *p* < 0.0001.

The detoxification repair agent is composed of two parts: mesenchymal stem cell exosomes and TDN. We first successfully extracted and identified bone marrow mesenchymal stem cells (BMSCs) (Figure S1, Supporting Information). The exosomes were then isolated and purified from the medium of the BMSCs. Transmission electron microscopy (TEM) and nanosight analysis (NTA) results showed that the exosomes were spherical vesicles with a particle size of 100 nm (**Figure** **1**A; Figure S2, Supporting Information). Meanwhile, western blot results confirmed the presence of specific proteins TSG101, CD81, and CD63, indicating that the extracted vesicles belonged to the exosome (Figure 1B). Next, we synthesized cholesterol‐modified TDNs and grafted them onto the exosomes. Agarose gel electrophoresis showed the successful synthesis of TDN (Figure 1C). Dynamic light scattering (DLS) and TEM results showed that the particle size of the TDN was ≈10 nm (Figures S3 and S4, Supporting Information). Atomic Force Microscopy (AFM) results showed that the height of the TDN was ≈2.5 nm (Figure 1D). As shown in Figure S5 (Supporting Information), even after adding TDN to a medium containing 10% serum, the agarose gel band was still present after 24 h, indicating the stability of TDN. The TDN was then modified on the surface of the exosome (Exo‐TDN). Exo did not generate a band in the agarose electrophoresis experiment, while TDN did. Thus, the agarose electrophoresis result could demonstrate successful loading of TDN (Figure 1E). The difference in the migration rates of TDN and Exo‐TDN was mainly due to the slow migration of exosomes with large molecular weights. Furthermore, immunogold staining followed by TEM imaging provided direct visual evidence that TDN was present on the Exo (Figure 1F). NTA and zeta potential analysis also proved the successful modification of TDN on exosomes (Figure 1G,H). When Exo and TDN reacted with a mass ratio of 1:3, the grafting efficiency of TDN reached the maximum rate of 66.2% (Figure 1I).

### 2.2 Exo‐TDN Captured DOX and Protected Cells

**Figure 2:**
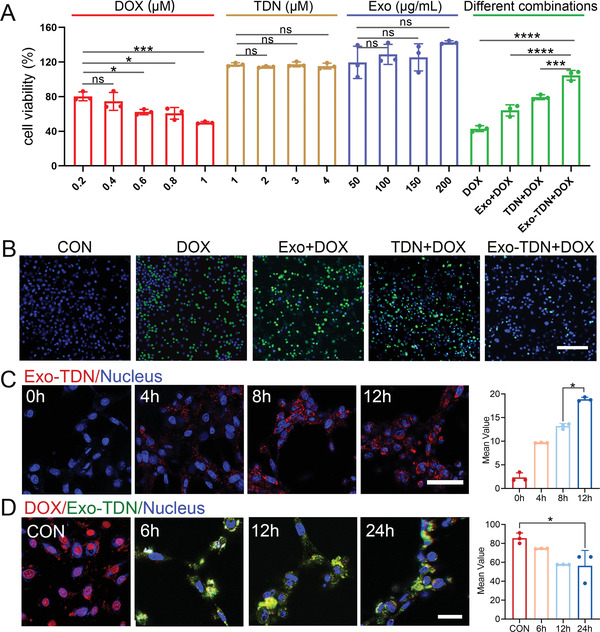
Exo‐TDN inhibited DOX‐induced apoptosis. A) The cell survival rates of BRL‐3A cell when treated with different concentrations of DOX, TDN, Exo, and Exo‐TDN (*n* = 3). B) LIVE–DEAD cell‐staining of BRL‐3A cell after treating with DOX, Exo+DOX, TDN+DOX, and Exo‐TDN +DOX (Blue: Live, Green: Dead). Scale bars, 100 µm. C) CLSM imaging of BRL‐3A cell uptake Exo‐TDN, and fluorescence quantification of Exo‐TDN in cytoplasm (*n* = 3). Scale bars, 50 µm. D) CLSM imaging of the inhibition of DOX from entering the nucleus by Exo‐TDN, as well as fluorescence quantification of DOX in the nucleus (*n* = 3). Scale bars, 50 µm. Data are presented as the mean ± SD. *p < 0.05; ^**^ *p* < 0.01; ^***^ *p* < 0.001; ^****^ *p* < 0.0001.

To prove the detoxification effect of Exo‐TDN, the capture efficiency of Exo‐TDN to DOX was first studied. According to previous reports, DOX is embedded in a “G‐C” base pair of double‐stranded DNA that quenches its fluorescence. As shown in Figure 1J, the introduction of Exo‐TDN significantly reduced the fluorescence intensity of DOX and the capture efficiency of Exo‐TDN reached up to 80% (Figure 1K). Next, we used the MTT method to verify the killing effect of DOX on cancer cells and their toxicity to normal cells. The results indicated that DOX showed significant proliferation inhibition effects on BRL‐3A cells (**Figure** **2**A). Next, we verified the biocompatibility of Exo and TDN. The result showed that Exo exhibited a certain promotional effect on cell proliferation (Figure 2A). Meanwhile, various concentrations of TDN showed no significant toxic effect on cells (Figure 2A). However, before administering chemotherapy drugs, the co‐incubation of cells with TDN, Exo and Exo‐TDN effectively reduced the toxicity of DOX (Figure 2A; Figure S6, Supporting Information). The LIVE–DEAD cell‐staining results also proved that the introduction of Exo‐TDN effectively inhibited apoptosis caused by DOX (Figure 2B; Figure S7, Supporting Information). To further explore the mechanism of detoxification, we observed the distribution of Exo‐TDN and DOX within cells through a confocal laser microscope (CLSM). The CLSM and flow cytometry (FC) results showed that Exo‐TDN was taken up by BRL‐3A cells and stably present in the cytoplasm (Figure 2C; Figure S8, Supporting Information). It is known that DOX inhibits cell replication and induces apoptosis by entering the nucleus and binding to DNA. The CLSM results confirmed the enrichment of free DOX in the nucleus (Figure 2D). However, Exo‐TDN in cytoplasm efficiently bound to DOX and prevented it from entering the nucleus, thereby further protecting cells from DOX damage (Figure 2D).

### 2.3 Anti‐Inflammatory Effects of Exo‐TDN

**Figure 3:**
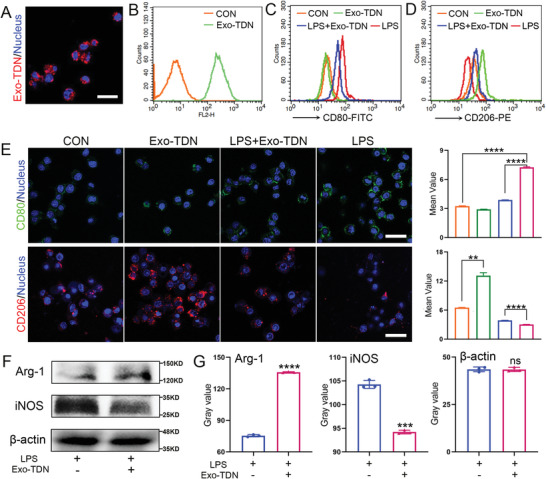
Exo‐TDN polarized macrophages to the M2 phenotype. A) CLSM imaging and B) FC results of RAW 264.7 cell uptake of Exo‐TDN. Scale bars, 20 µm. C,D) FC results of the expression of CD80 (C) and CD206 (D) in RAW 264.7 cells. E) CLSM imaging and fluorescence quantification of CD80 and CD206 expression in RAW 264.7 cells (*n* = 3). Scale bars, 20 µm. F) Western blot results of the expression of Arg‐1 and iNOS in RAW 264.7 cells. G) Grayscale statistics for different protein bands (*n* = 3). Data are presented as the mean ± SD. *p < 0.05; ^**^ *p* < 0.01; ^***^ *p* < 0.001; ^****^ *p* < 0.0001.

Since eliminating inflammation promotes healing of damaged tissue, we further studied the anti‐inflammatory effects of Exo‐TDN. CLSM and FC results showed that Exo‐TDN was taken up by macrophages (**Figure** **3**A,B). Using CLSM and FC analysis, we evaluated the expression of two macrophage markers, CD80 (M1 marker) and CD206 (M2 marker), after incubation with exosomes. Compared to the control group or the LPS‐treated group, the Exo‐TDN group showed a lower expression of CD80 and higher expression of CD206 after 24 h treatment (Figure 3C–E). In addition, we analyzed the expression of intracellular proteins iNOS and Arg‐1 in macrophages. The western blot results showed higher expression of iNOS but lower expression of Arg‐1 compared to the control group, indicating that Exo‐TDN polarized M0 or M1 macrophages to the M2 phenotype (Figure 3F,G). These results confirmed that exosomes played an anti‐inflammatory role, which would further promote the repair of damaged tissue.

### 2.4 The Biodistribution of Exo‐TDN or Exo‐TDN‐PCM In Vivo

**Figure 4:**
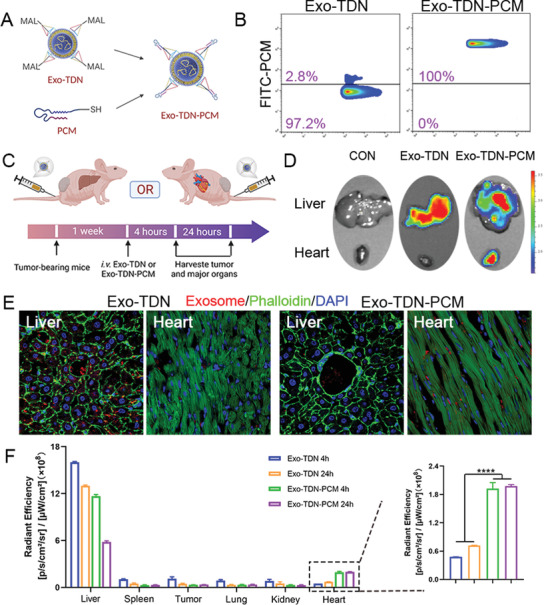
The biodistribution of Exo‐TDN or Exo‐TDN‐PCM in tumor‐bearing mice. A) Schematic of the synthesis of Exo‐TDN‐PCM. B) Nano‐flow cytometry results of Exo‐TDN and Exo‐TDN‐PCM. C) Schematic of the in vivo distribution experiment to test targeting capabilities of Exo‐TDN or Exo‐TDN‐PCM. D) Ex vivo fluorescence imaging to detect the biodistribution of DiD‐labeled Exo‐TDN and Exo‐TDN‐PCM after 4 h of in vivo injection. E) CLSM images showing the distribution of DiD‐labeled Exo‐TDN and Exo‐TDN‐PCM. F) Quantitative analysis of fluorescent intensity in the different tissues after injection of Exo‐TDN or Exo‐TDN‐PCM for 4 and 24 h (*n* = 3). Data are presented as the mean ± SD. *p < 0.05; ^**^ *p* < 0.01; ^***^ *p* < 0.001; ^****^ *p* < 0.0001.

By grafting the target peptides on the TDN, the multi‐organ applicability of the detoxification repair agent can be expanded. We first synthesized a maleimide‐including TDN and engineered it on the Exo surface (Exo‐TDN‐MAL). Cysteine‐modified cardiomyopathic peptide PCM (Cys‐PCM) was grafted on the surface of Exo‐TDN‐MAL through a click reaction, generating the heart detoxification repair agent of Exo‐TDN‐PCM (**Figure** **4**A). As shown in Figure 4B, the nano‐flow cytometry result showed that Cys‐PCM‐FITC was successfully grafted on Exo‐TDN. Furthermore, modifying the PCM did not affect capture efficiency of Exo‐TDN to DOX, which prevented it from entering the nucleus (Figure S9, Supporting Information). Next, we i.v. injected DiD (1,1“‐Dioctadecyl‐3,3,3”,3'‐Tetramethylindodicarbocyanine,4‐Chlorobenzenesulfonate Salt)‐labeled Exo‐TDN or Exo‐TDN‐PCM, and then use the small animal in vivo imaging system (IVIS) to verify their targeting capabilities (Figure 4C). As shown in Figure 4D, 4 h post‐injection, Exo‐TDN was completely gathered in the liver area, while Exo‐TDN‐PCM was partially gathered in the myocardial area. We also observed the corresponding tissues using CLSM to provide additional evidence for the distribution of Exo‐TDN and Exo‐TDN‐PCM (Figure 4E; Figure S10, Supporting Information). Exo‐TDN or Exo‐TDN‐PCM was still entirely accumulated in the liver or heart area 24 h post‐injection. They mainly failed to reach the tumor area, thus indicating that they would not adsorb DOX in tumor tissue and would have no effect on the tumor‐killing effect of DOX (Figure 4F; Figure S11, Supporting Information). Furthermore, the results of Figure 4F also demonstrated that modifying PCM could significantly increase the enrichment of Exo‐TDN‐PCM to the heart.

### 2.5 Study of Liver Protection by Exo‐TDN In Vivo

**Figure 5:**
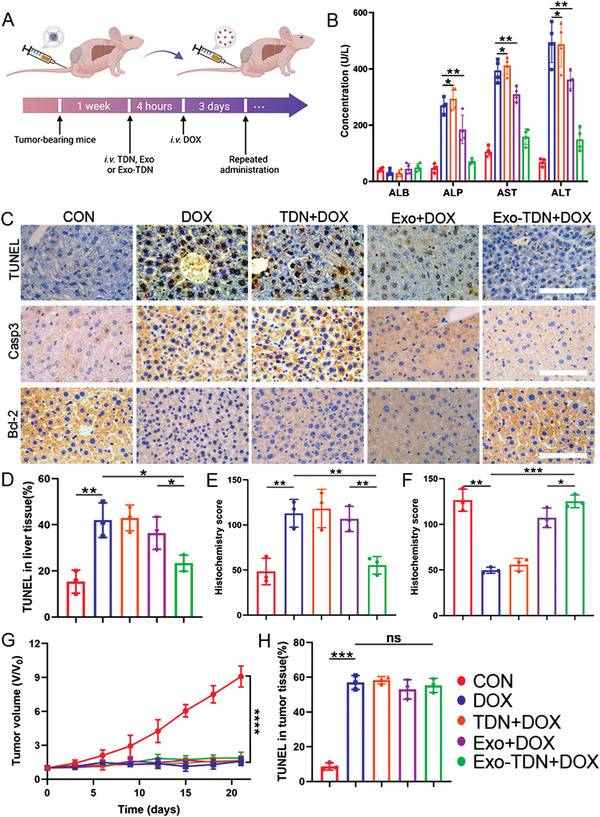
Study of liver protection effect of Exo‐TDN. A) Schematic of the overall design of the animal experiments to test the liver protection of Exo‐TDN. B) Blood biochemical analysis of mice after 21 days of treatment (*n* = 4). C) TUNEL and apoptosis‐related proteins staining analysis of liver tissue in the treatment of different groups. Scale bars, 100 µm. D–F) Quantitative analysis of TUNEL staining images (D), Casp3 staining images (E), and Bcl‐2 staining images (F) (*n* = 3). G) Tumor volume changes of mice in different treatment groups (*n* = 8). H) Quantitative analysis of TUNEL staining images of tumor tissue in the treatment of different groups (*n* = 3). Data are presented as the mean ± SD. *p < 0.05; ^**^ *p* < 0.01; ^***^ *p* < 0.001; ^****^ *p* < 0.0001.

To further demonstrate the detoxification effect of Exo‐TDN, the MCF‐7 tumor‐bearing mice model was constructed and subsequently chemotherapy was performed (**Figure** **5**A). After 21 days of treatment, we collected blood from different groups and conducted blood biochemical tests. The results showed that enzymes associated with liver damage, such as ALP, AST, and ALT, were significantly reduced (Figure 5B; Table S1, Supporting Information). Subsequently, we collected liver tissue from mice and performed pathological analysis. H&E and Masson staining results showed that DOX‐induced liver damage was significantly reduced in the Exo‐TDN group compared to the control group and other therapeutic groups (Figures S12 and S13, Supporting Information). Furthermore, we also performed immunohistochemical (IHC) analysis to study the liver‐protecting effect (Figure 5C). TUNEL staining results showed that Exo‐TDN significantly reduced DOX‐induced liver cell apoptosis compared to other groups (Figure 5D; Figure S14, Supporting Information). Furthermore, the liver protection mechanism of Exo‐TDN was verified by analyzing apoptosis‐related proteins Casp3 and Bcl‐2. IHC results showed that Exo‐TDN effectively attenuated the expression of apoptosis protein Casp3 and increased the expression of the anti‐apoptotic protein Bcl‐2, thus reducing DOX‐induced hepatocellular apoptosis, which was consistent with our in vitro results described above (Figure 5E,F; Figure S14, Supporting Information).

It was important to study if our Exo‐TDN had an effect on DOX‐induced cancer therapy. We verified the effect of Exo‐TDN on DOX‐induced tumor growth inhibition. The results of tumor volume change showed that the injection of DOX effectively inhibited tumor growth compared to the control group (Figure 5G; Figure S15, Supporting Information). After 21 days of treatment, we collected tumor tissue and performed pathological analysis. The results showed that DOX effectively promoted tumor apoptosis, indicating that the introduction of Exo, TDN, or Exo‐TDN did not affect the killing effect of DOX (Figure 5H; Figure S16, Supporting Information). In addition, we monitored and recorded the weight of the mice. Compared to the DOX, Exo+DOX, and TDN+DOX groups, the Exo‐TDN+DOX group showed a slow increase in mouse weight (Figure S17, Supporting Information). This result proved that the introduction of Exo‐TDN reduced the toxic effects of chemotherapy drugs.

### 2.6 Study of Heart Protection of Exo‐TDN‐PCM

**Figure 6:**
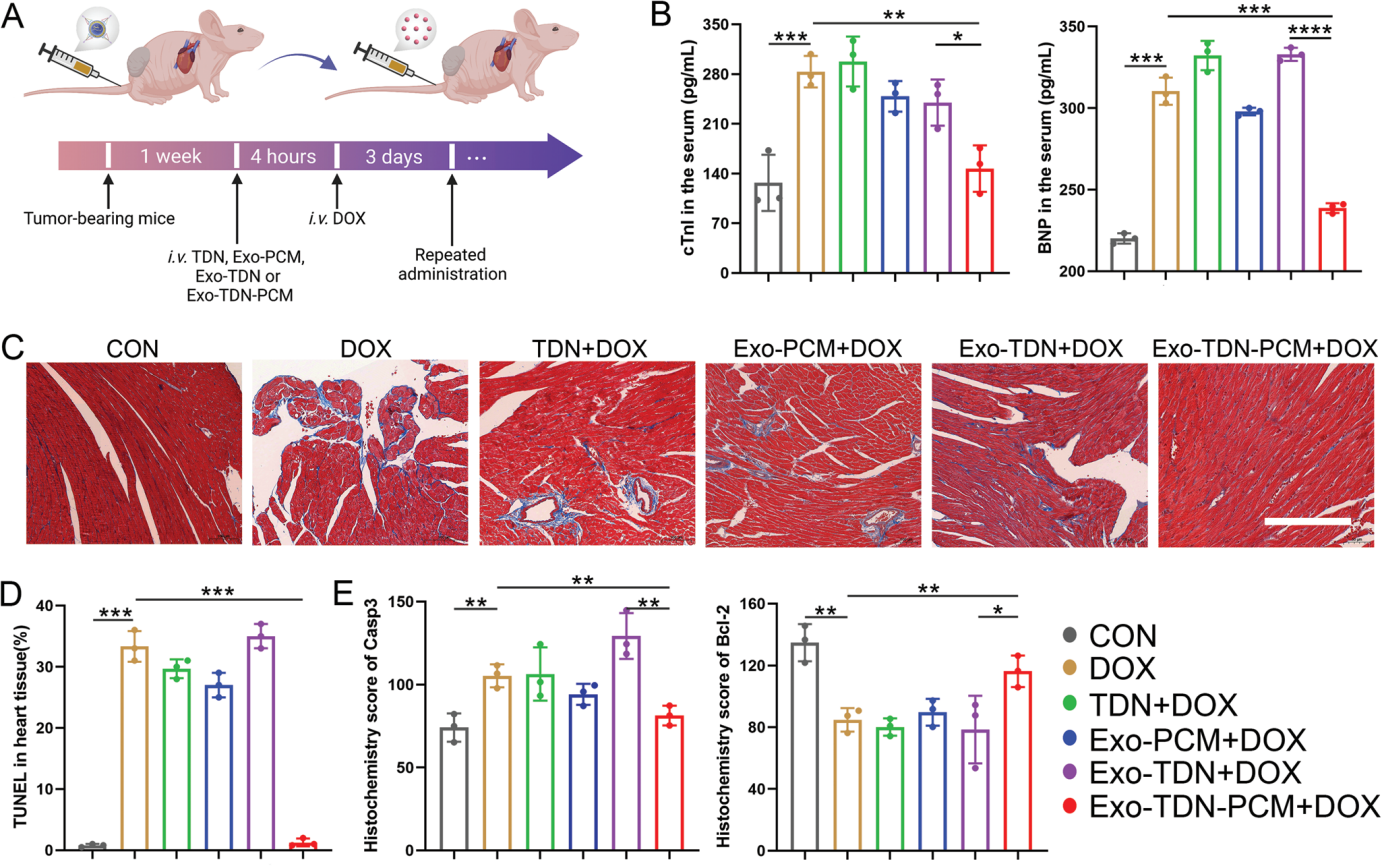
The study of heart protection of Exo‐TDN‐PCM. A) Schematic of the overall design of the animal experiments to test the heart protection of Exo‐TDN‐PCM. B) The content of BNP and cTnI in serum after 42 days of treatment (*n* = 3). C) Masson staining images of heart tissue in different treatment groups. Scale bars, 100 µm. D) Quantitative analysis of TUNEL staining images (*n* = 3). E) Quantitative analysis of immunohistochemical staining images of heart tissue showing, Casp3, Bcl‐2 expression (*n* = 3). Data are presented as the mean ± SD. *p < 0.05; ^**^ *p* < 0.01; ^***^ *p* < 0.001; ^****^ *p* < 0.0001.

After successfully demonstrating the liver‐protecting effect of Exo‐TDN and its slight effect on chemotherapy, we next broadened their application in relieving DOX‐induced cardiotoxicity by engineering Exo‐TDN with a myocardium‐targeting peptide (**Figure** **6**A). DOX‐induced cardiotoxicity has been widely known to result in the death of cancer patients. We first detected the serum markers of myocardial injury after treatment. We collected blood from different groups and detected the content of BNP and cTnI in serum. The results showed that the content of BNP and cTnI in the Exo‐TDN‐PCM group was significantly reduced after 42 days of treatment, indicating the reduction of DOX‐induced cardiotoxicity (Figure 6B). Subsequently, we collected heart tissue and performed pathological analysis. Both Masson's trichrome staining and H&E staining results showed that the damage to heart tissue caused by DOX was significantly attenuated in the Exo‐TDN‐PCM group compared to the control group and other therapeutic groups (Figure 6C; Figures S18 and S19, Supporting Information). In addition, TUNEL staining results showed that Exo‐TDN‐PCM could effectively mitigate the DOX‐caused heart toxicity (Figure 6D; Figure S20, Supporting Information).

### 2.7 Heart Protection Mechanism of Exo‐TDN‐PCM

**Figure 7:**
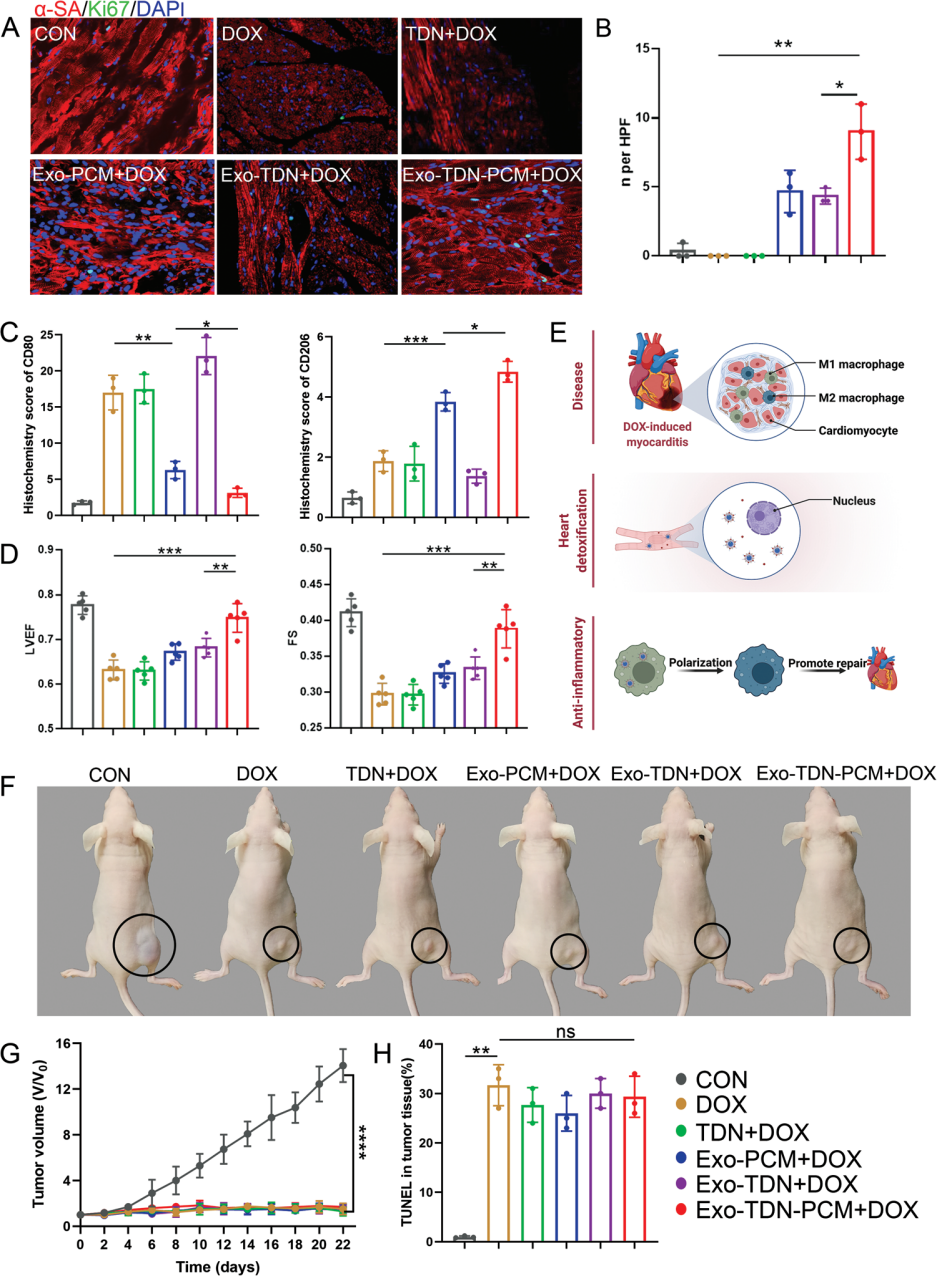
The study of heart protection mechanisms of Exo‐TDN‐PCM. A) CLSM images of Ki67 staining of heart sections 42 days after injection. Scale bars, 100 µm. B) Quantitative data corresponding to Ki67 staining (*n* = 3). *n* per HPF is a unit of fluorescence brightness. *n* refers to the quantified fluorescence intensity. HPF refers to the high power field. C) Histochemistry score of immunohistochemical staining images of CD80 and CD206 (*n* = 3). D) Left ventricular ejection fraction (LVEF) and fractional shortening (FS) 42 days after treatment (*n* = 5). E) Schematic of the heart protection mechanism of Exo‐TDN‐PCM. F) Representative photographs of tumor after 22 days of treatment. G) Tumor volume changes of mice in different treatment groups (*n* = 6). H) Histochemistry score of TUNEL staining images of tumor tissue in the treatment of different groups (*n* = 3). Data are presented as the mean ± SD. *p < 0.05; ^**^ *p* < 0.01; ^***^ *p* < 0.001; ^****^ *p* < 0.0001.

Furthermore, the heart protection mechanism of Exo‐TDN‐PCM was verified. Immunostaining of apoptosis‐related proteins showed that Exo‐TDN‐PCM effectively reduced myocardial apoptosis, thereby reducing DOX‐induced myocardial damage (Figure 6E; Figure S21, Supporting Information). Subsequently, we evaluated the ability of Exo‐TDN‐PCM to promote myocardial tissue repair. After injection of Exo‐TDN‐PCM, the number of Ki67‐positive cells was significantly improved in myocardial tissue (**Figure** **7**A,B). In addition, we analyzed the infiltration and phenotype of macrophages in myocardial tissue by immunohistochemical staining. The results showed lower expression of CD80 and higher expression of CD206 in the Exo‐TDN‐PCM group (Figure 7C; Figure S22, Supporting Information). The results indicated that Exo‐TDN‐PCM could effectively reduce inflammation of myocardial tissue by promoting the M1 to M2 transition of macrophages. The reduction of inflammation could further promote the repair of myocardial tissue. In line with the morphological benefits, injection of Exo‐TDN‐PCM augmented left ventricular ejection fraction (LVEF) and fractional shortening (FS) (Figure 7D). In short, Exo‐TDN‐PCM could effectively reduce myocardial apoptosis, as well as relieve inflammation of myocardial tissue and further promote tissue repair, thereby reducing DOX‐induced cardiomyopathy (Figure 7E). We then verified the effect of Exo‐TDN‐PCM on DOX‐induced tumor growth inhibition. The results of tumor volume change and TUNEL staining showed that the injection of DOX effectively inhibited tumor growth compared to the control group (Figure 7F–H; Figure S23, Supporting Information).

## 3 Conclusion

In summary, we synthesized Exo‐TDN for detoxification and repair of major organs. We first extracted rat BMSCs and characterized them by FC and differentiation induction. We then extracted BMSC exosomes and functionalized them with tetrahedral DNA abundant in G‐C bases. FC and CLSM results proved that Exo‐TDN could indeed capture DOX in cells and prevented it from entering the nucleus. In this work, organ damage caused by DOX during cancer treatment was reversed by synthesis of tetrahedral DNA engineered exosomes. Tumor‐bearing mice were first protected by our Exo‐TDN before administrating DOX for chemotherapy. Our in vivo experiments showed that Exo‐TDN was effectively enriched in liver tissue and significantly reduced DOX‐induced liver damage while having only a slight effect on DOX‐induced chemotherapy. Furthermore, chemotherapy‐related cardiac dysfunction is known as one of the most notorious short‐term side effects of anticancer treatment. To relieve DOX‐induced cardiotoxicity, we further structured a detoxification repair agent to attenuate cardiotoxicity by modifying heart‐targeting peptides on the surface of Exo‐TDN. Exo‐TDN‐PCM had been shown to significantly reduce DOX‐induced cardiotoxicity through anti‐apoptosis effects. Moreover, as exosomes have been widely used for injured tissue repair, we have demonstrated that our Exo‐TDN‐PCM showed enhanced cardiomyocyte proliferation and anti‐inflammatory effects. Therefore, we believe that Exo‐TDN can be used to detoxify and repair multiple organs, and thus improve the survival time of cancer patients.

## 4 Experimental Section

### Isolation of Bone Marrow Mesenchymal Stem Cells (BMSCs)

Three‐week‐old rats were sacrificed by dislocation; the femur and tibia were taken out using forceps and scissors, after which they were placed in PBS containing 1% penicillin and streptomycin. The epiphyses at both ends of the femur and tibia were excised in PBS to completely allow leaking from the bone cavity, after which they were placed in DMEM containing 10% FBS and 1% penicillin and streptomycin. A 1 mL syringe was used to flush out the bone marrow with culture medium. The bone marrow was then transferred to a culture flask and placed in an incubator at 37 °C containing 5% CO_2_. After 3 days of cultivation, the medium was changed. The cells obtained at this time were P0 generation cells.^[^ ^24^ ^]^ Every 2 days, the medium was discarded and the cells were washed with PBS and fresh medium was added. After the cell confluence reached 80–90%, they were digested with trypsin and then sub‐cultured. P3 generation cells were selected to identify mesenchymal stem cells, and P3‐P5 generation BMSCs were selected to extract exosomes.

### Exosome Extraction

When the confluence of P3‐P5 BMSCs reached 70–80%, the medium was removed and replaced with a serum‐free medium. After culturing for 12 h, the supernatant was collected and centrifuged at 10000 rpm for 30 min. Cell debris was then removed, after which a 0.22 µm filter was used to remove large, extracellular vesicles. Then, the steps in the exosome extraction kit instructions were followed: 1/3 of the volume of the cell culture supernatant exosome extraction reagent was added, followed by 4 °C incubation overnight and then centrifuging at 10 000 × *g* for 30 min at 4 °C. Finally, the resulting pellet was obtained, resuspended in PBS, and stored at −80 °C.^[^ ^25^ ^]^

### The Synthesis of DNA with Different Structures

S1‐cho‐two strands (S1‐cho and S2‐cho), three strands (S1‐cho, S2‐cho and S3‐cho), and four strands (S1‐cho, S2‐cho, S3‐cho and S4) in equimolar amounts (for a final concentration of 2 × 10^−6^ m) was mixed in a buffer (20 × 10^−3^ m Tris and 16 × 10^−3^ m MgCl_2_ at a pH of 8.0) then kept at 95 °C for 10 min, after which it was slowly cooled to 4 °C. Different structures of DNA were obtained, in which equimolar amounts of four‐stranded strands were reacted to obtain TDN.^[^ ^26^ ^]^ The sequence of each single strand of DNA is shown in Table 1.

**Table 1:** The sequence of each single strand of DNA

ssDNA	Sequence (5“‐3”)
S1	GAGCGTTAGCCACACACACAGTC
S2	TTAGGCGAGTGTGGCAGAGGTGT
S3	CGCCTAAACAAGTGGAGACTGTG
S4	AACGCTCACCACTTGAACACCTC

### The Stability of Tetrahedral DNA

To verify the stability of the tetrahedral DNA, synthetic tetrahedral DNA was added to 1640 medium (10% FBS) and incubated at 37 °C multiple times. Then, 10 µL was taken, into which 2 µL of 6 × DNA loading buffer was added. The samples were mixed well and then loaded into the gel well, where they were run at 90 V for 30 min. Then, a gel imager was used to take pictures.

### The Synthesis of Exo‐TDN

A total of 10 µg of exosomes was taken and dispersed into PBS, after which 30 µg of the aforementioned synthetic tetrahedral DNA was added. The samples were incubated at 37 °C for 1 h and then quickly put on ice and shaken for 1 h. Then, a 100 KDa ultrafiltration tube was used to centrifuge the samples at 4500 × *g* for 15 min to remove excess tetrahedral DNA, after which exosomes engineered with tetrahedral DNA were obtained.^[^ ^27^ ^]^

### Exo‐TDN Captures DOX In Vitro

To verify the ability of Exo‐TDN to capture DOX, 1 × 10^−6^ m DOX was incubated with increasing concentrations of Exo‐TDN at 37 °C for 1 h, after which the fluorescence change at a wavelength of 480 nm was measured under maximum excitation. The captured DOX was then calculated from the standard DOX curve.

### The Synthesis of Exo‐TDN‐PCM

First, a maleimide‐including TDN synthesized and engineered on the Exo surface (Exo‐TDN‐MAL). An appropriate amount of cysteine‐modified cardiomyopathic peptide PCM was added to the Exo‐TDN‐MAL solution. After continuous oscillation at 4 °C for 12 h, the heart detoxification repair agent EXO‐TDN‐PCM was obtained.

According to the previous method,^[^ ^26^, ^27^, ^28^, ^29^ ^]^ Exo‐PCM acted as a control group. An appropriate amount of DSPE‐PEG‐MAL was added to the Cys‐PCM solution. After continuous oscillation at 4 °C for 24 h, the DSPE‐modified PCM (DSPE‐PEG‐PCM) was obtained. Subsequently, an appropriate amount of DSPE‐PEG‐PCM was added to the Exo solution. After continuous oscillation at 4 °C for 4 h, the PCM‐modified Exo (Exo‐PCM) was obtained.

### Cell Protection of Exo‐TDN

MTT methods were used to evaluate the protective effect of the materials on the cells. In a 96‐well plate, BRL‐3A cells were seeded at a density of 1 × 10^3^–2 × 10^3^ cells per well. They were then incubated overnight to make the cells adhere to the well. Different concentrations of TDN and Exo were added to the cell plate. After 4 h, DOX was added to make the final concentration 0.2–1 × 10^−6^ m. MTT was added after 24 h of incubation in an incubator at 37 °C and 5% CO_2_. Absorbance was then detected by a microplate reader, after which the survival rates of different groups of cells were calculated.

In addition, LIVE–DEAD cell staining was used to analyze cell protection of Exo‐TDN. Cells were seeded in a 48‐well plate at a density of 1 × 10^4^ cells per well and cultured overnight. Then, 50 µL of PBS, Exo (60 µg), TDN (60 µg), or Exo‐TDN (60 µg) were added for 4 h of incubation, after which 2 × 10^−6^ m of DOX was added, followed by another 24 h of incubation. Then, one drop of NucBlue Live reagent and NucGreen Dead reagent was added to each well, followed by incubation for 20 min. Finally, the dye was washed away with PBS, and the samples were transferred to a fluorescence microscope for observation.

### Exo‐TDN Inhibited DOX from Entering Nuclei

The BRL‐3A cells were inoculated in a 30 mm glass‐bottom culture dish at a density of 1 × 10^4^ cells per well. After the cells adhered to the bottom, 60 µg of Exo‐TDN was added, followed by 4 h of incubation. Then, 2 × 10^−6^ m of DOX was added, followed by another 6, 12, or 24 h of incubation. The medium was cultured and washed three times with PBS, after which the nuclear dye Hoechst 33 342 was added, followed by incubation for 10 min. This was followed by three washings with PBS. The samples were then observed via CLSM. Three parallel wells were set up at each time point. Three fields of view were selected for shooting in each well, and then the average of red fluorescence intensity of the nuclei in the three fields of view was calculated. Finally, the average of the three wells was counted to get a histogram.

### Heart Protection Model

MCF‐7 cells were digested with trypsin, washed twice with PBS and resuspended with Matrigel in equal volume. The sample density was adjusted to 1 × 10^7^ cell per mL, and 0.1 mL was inoculated under the skin of the right back of the nude mice. When the tumors reached ≈70–80 mm^3^, the mice were divided into six groups with six tumor‐bearing mice in each group. The experimental groups were the control group, the DOX group, the TDN+DOX group, the Exo‐PCM+DOX group, the Exo‐TDN+DOX group, and the Exo‐TDN‐PCM+DOX group. The control group was intravenously injected with 500 µL of PBS, and received a second injection of 100 µL of PBS 4 h later. The mice in the DOX group were treated with 500 µL of PBS, and 4 h later they were given 100 µL of DOX (5 mg kg^−1^) injected into the tail vein. In the TDN+DOX group, the mice were intravenously injected with 500 µL of PBS containing 20 µg of TDN, and then they received a second injection of 100 µL of DOX (5 mg kg^−1^) 4 h later. In the Exo‐PCM + DOX, Exo‐TDN + DOX, and Exo‐TDN‐PCM + DOX group, mice were injected intravenously with 500 µL of PBS containing 20 µg of Exo‐PCM, Exo‐TDN, or Exo‐TDN‐PCM, respectively, after which they received a second tail vein injection of 100 µL of DOX (5 mg kg^−1^) 4 h later.^[^ ^30^ ^]^ This treatment was repeated every 3 days, and tumor sizes were measured with a caliper and mice were weighed. Cardiac function was measured by using the Vevo 2100 Imaging System. Both ejection fraction (EF) and fractional shortening (FS) were determined by measurements from the left ventricular internal diameter at end‐diastole (LVIDd) and systole (LVIDs). After 42 days, mouse myocardial tissue was collected and subjected to pathological and immunomic analysis. Subsequently, immunohistochemical images were quantified through Aipathwell. Histochemistry score (H‐Score) was used to represent semi‐quantitative results of tissue staining for each slice. H‐Score = ∑(pi × i) = (percentage of weak intensity × 1) + (percentage of moderate intensity × 2) + (percentage of strong intensity × 3).^[^ ^31^, ^32^ ^]^

## Conflict of Interest

The authors declare no conflict of interest.

## Supporting information

Supporting Information

## Data Availability

Research data are not shared.

## References

[1] B. W. Ward , J. S. Schiller , R. A. Goodman , Prev. Chronic Dis. 2014, 11, 62. 10.5888/pcd11.130389 PMC3992293 24742395

[2] H. Sung , J. Ferlay , R. L. Siegel , M. Laversanne , I. Soerjomataram , A. Jemal , F. Bray , CA Cancer J. Clin. 2021, 71, 209. 33538338 10.3322/caac.21660

[3] R. Zhao , X. Liu , X. Yang , B. Jin , C. Shao , W. Kang , R. Tang , Adv. Mater. 2018, 30, 1801304. 10.1002/adma.201801304 29761566

[4] D. E. Kleiner , N. P. Chalasani , W. M. Lee , R. J. Fontana , H. L. Bonkovsky , P. B. Watkins , P. H. Hayashi , T. J. Davern , V. Navarro , R. Reddy , J. A. Talwalkar , A. Stolz , J. Gu , H. Barnhart , J. H. Hoofnagle , Hepatology 2014, 59, 661. 24037963 10.1002/hep.26709 PMC3946736

[5] L. Li , G. Takemura , Y. Li , S. Miyata , M. Esaki , H. Okada , H. Kanamori , N. C. Khai , R. Maruyama , A. Ogino , S. Minatoguchi , T. Fujiwara , H. Fujiwara , Circulation 2006, 113, 535. 16449733 10.1161/CIRCULATIONAHA.105.568402

[6] D. N. Waterhouse , P. G. Tardi , L. D. Mayer , M. B. Bally , Drug Saf. 2001, 24, 903. 11735647 10.2165/00002018-200124120-00004

[7] Z. Z. Wang , K. D. Popowski , D. S. Zhu , B. L. de Juan Abad , X. Y. Wang , M. R. Liu , H. Lutz , N. De Naeyer , C. T. DeMarco , T. N. Denny , P. C. Dinh , Z. H. Li , K. Cheng , Nat. Biomed. Eng. 2022, 6, 791. 35788687 10.1038/s41551-022-00902-5 PMC10782831

[8] K. D. Popowski , B. L. de Juan Abad , A. George , D. Silkstone , E. Belcher , J. Chung , A. Ghodsi , H. Lutz , J. Davenport , M. Flanagan , J. Piedrahita , P. C. Dinh , K. Cheng , Extracellular Vesicle 2022, 1, 100002. 10.1016/j.vesic.2022.100002 PMC9213043 36523538

[9] Z. H. Li , Z. Z. Wang , P. C. Dinh , D. S. Zhu , K. D. Popowski , H. Lutz , S. Q. Hu , M. G. Lewis , A. Cook , H. Andersen , J. Greenhouse , L. Pessaint , L. J. Lobo , K. Cheng , Nat. Nanotechnol. 2021, 16, 942. 34140674 10.1038/s41565-021-00923-2 PMC8364483

[10] Y. Wen , Q. Fu , A. Soliwoda , S. Zhang , M. F. Zheng , W. J. Mao , Y. Wan , Extracellular Vesicle 2022, 1, 100004. 10.1016/j.vesic.2022.100004 PMC9794200 36578271

[11] R. Molinaro , C. Corbo , J. O. Martinez , F. Taraballi , M. Evangelopoulos , S. Minardi , I. K. Yazdi , P. Zhao , E. De Rosa , M. B. Sherman , A. De Vita , N. E. Toledano Furman , X. Wang , A. Parodi , E. Tasciotti , Nat. Mater. 2016, 15, 1037. 27213956 10.1038/nmat4644 PMC5127392

[12] S. Hu , Z. Li , D. Shen , D. Zhu , K. Huang , T. Su , P.‐U. Dinh , J. Cores , K. Cheng , Nat. Biomed. Eng. 2021, 5, 1174. 33820981 10.1038/s41551-021-00705-0 PMC8490494

[13] M. Liu , H. Lutz , D. Zhu , K. Huang , Z. Li , P. C. Dinh , J. Gao , Y. Zhang , K. Cheng , Adv. Sci. 2020, 8, 2002127. 10.1002/advs.202002127 PMC7788635 33437573

[14] Y. Zhang , L. Li , J. Yu , D. Zhu , Y. Zhang , X. Li , H. Gu , C. Y. Zhang , K. Zen , Biomaterials 2014, 35, 4390. 24565517 10.1016/j.biomaterials.2014.02.003

[15] L. Alvarez‐Erviti , Y. Seow , H. Yin , C. Betts , S. Lakhal , M. J. Wood , Nat. Biotechnol. 2011, 29, 341. 21423189 10.1038/nbt.1807

[16] J. G. van den Boorn , J. Dassler , C. Coch , M. Schlee , G. Hartmann , Adv. Drug Delivery Rev. 2013, 65, 331. 10.1016/j.addr.2012.06.011 22750807

[17] T. A. Wynn , A. Chawla , J. W. Pollard , Nature 2013, 496, 445. 23619691 10.1038/nature12034 PMC3725458

[18] L. C. Davies , S. J. Jenkins , J. E. Allen , P. R. Taylor , Nat. Immunol. 2013, 14, 986. 24048120 10.1038/ni.2705 PMC4045180

[19] C. Zhao , H. Song , P. Scott , A. Zhao , H. Tateishi‐Karimata , N. Sugimoto , J. Ren , X. Qu , Angew. Chem. Int. Ed. Engl. 2018, 57, 15723. 30311333 10.1002/anie.201809207

[20] S. Q. Hu , Z. H. Li , D. L. Shen , D. S. Zhu , K. Huang , T. Su , P.‐U. Dinh , J. Cores , K. Cheng , Nat. Biomed. Eng. 2021, 5, 1174. 33820981 10.1038/s41551-021-00705-0 PMC8490494

[21] J. L. Yao , K. Huang , D. S. Zhu , T. Chen , Y. F. Jiang , J. Y. Zhang , L. J. Mi , H. Xuan , S. Q. Hu , J. L. Li , Y. F. Zhou , K. Cheng , ACS Nano 2021, 15, 11099. 34152126 10.1021/acsnano.1c00628

[22] N. Su , Y. Y. Hao , F. Wang , W. D. Hou , H. F. Chen , Y. Luo , Sci. Adv. 2021, 7, 7207. 10.1126/sciadv.abf7207 PMC8115917 33980490

[23] J. X. Zhao , X. L. Li , J. X. Hu , F. Chen , S. H. Qiao , X. Sun , L. Gao , J. Xie , B. Xu , Cardiovasc. Res. 2019, 115, 1205. 30753344 10.1093/cvr/cvz040 PMC6529919

[24] M. Soleimani , S. Nadri , Nat. Protoc. 2009, 4, 102. 19131962 10.1038/nprot.2008.221

[25] Q. Q. Cheng , X. J. Shi , M. L. Han , G. Smbatyan , H. Lenz , Y. Zhang , J. Am. Chem. Soc. 2018, 140, 16413. 30452238 10.1021/jacs.8b10047 PMC6469991

[26] J. Li , K. y. Xun , K. Pei , X. J. Liu , X. Y. Peng , Y. L. Du , L. P. Qiu , W. H. Tan , J. Am. Chem. Soc. 2019, 141, 18013. 31626550 10.1021/jacs.9b04725

[27] J. L. Zhuang , J. Z. Tan , C. L. Wu , J. Zhang , T. Liu , C. H. Fan , J. P. Li , Y. Q. Zhang , Nucleic Acids Res. 2020, 48, 8870. 32810272 10.1093/nar/gkaa683 PMC7498310

[28] Z. H. Li , S. Q. Hu , K. Huang , T. Su , J. Cores , K. Cheng , Sci. Adv. 2020, 6, 589. 10.1126/sciadv.aay0589 PMC7002120 32076644

[29] M. Fan , H. F. Liu , H. Y. Yan , R. J. Che , Y. Jin , X. J. Yang , X. H. Zhou , H. Yang , K. Ge , X.‐J. Liang , J. C. Zhang , Z. H. Li , Biomaterials 2022, 282, 121424. 35196606 10.1016/j.biomaterials.2022.121424

[30] R. B. Zhao , X. Y. Liu , X. Y. Yang , B. Jin , C. Y. Shao , W. J. Kang , R. K. Tang , Adv. Mater. 2018, 30, 1801304. 10.1002/adma.201801304 29761566

[31] R. Guo , L. D. Berry , D. L. Aisner , J. Sheren , T. Boyle , P. A. Bunn Jr. , B. E. Johnson , D. J. Kwiatkowski , A. Drilon , L. M. Sholl , M. G. Kris , J. Thorac. Oncol. 2019, 14, 1666. 31228623 10.1016/j.jtho.2019.06.009 PMC6708730

[32] A. Paschalis , B. Sheehan , R. Riisnaes , D. N. Rodrigues , G. B , C. Bertan , A. Ferreira , M. B. K. Lambros , G. Seed , W. Yuan , D. Dolling , J. C. Welti , A. Neeb , S. Sumanasuriya , P. Rescigno , D. Bianchini , N. Tunariu , S. Carreira , A. Sharp , W. Oyen , J. S de Bono. Eur. Urol. 2019, 76, 469. 10.1016/j.eururo.2019.06.030 PMC6853166 31345636

