# Supplementary material for: Decoy Exosomes Offer Protection Against Chemotherapy‐Induced Toxicity
Source: Adv Sci (Weinh). 2022 Sep 4;9(32):2203505. doi: 10.1002/advs.202203505 (PMC9661835; doi:10.1002/advs.202203505)
Supplement: Supplementary file 1 — Supporting Information [file ADVS-9-2203505-s001.pdf]

ADVANCED SCIENCE
Open Access

Supporting Information

for *Adv. Sci.*, DOI 10.1002/advs.202203505

Decoy Exosomes Offer Protection Against Chemotherapy-Induced Toxicity

Miao Fan, Hang Li, Deliang Shen, Zhaoshuo Wang, Huifang Liu, Dashuai Zhu, Zhenzhen Wang, Lanya Li, Kristen D. Popowski, Caiwen Ou, Kaihan Zhang, Jinchao Zhang, Ke Cheng* and Zhenhua Li**

Supporting Information

Decoy Exosomes Offer Protection Against Chemotherapy-Induced Toxicity

Miao Fan, Hang Li, Deliang Shen, Zhaoshuo Wang, Huifang Liu, Dashuai Zhu, Zhenzhen Wang, Lanya Li, Kristen D. Popowski, Caiwen Ou, kaihan Zhang, Jinchao Zhang, Ke Cheng*, Zhenhua Li**

Cell culture

The MCF-7 cell lines were cultured in DMEM medium containing 10% FBS and 1% penicillin-streptomycin. BRL-3A cell lines were cultured in 10% FBS1640 medium with 1% penicillin-streptomycin. The cells were cultured at 37 °C in 5% CO_2, and experiments were performed after the cells had entered the logarithmic growth phase.

The identification of the differentiation ability of bone marrow mesenchymal stem cells (BMSCs)

Alizarin Red staining: P3 BMSCs were seeded in a 24-well plate at a density of 1×10^5 cells/well. When the cell confluence reached about 60%, 1 mL of osteoinductive agent was added, and the medium was changed every three days for 21 days of culture. After the induction was complete, the medium was aspirated and cells were washed with PBS three times. Then, 95% ethanol was added for fixation for ten minutes, after which 0.1% Alizarin Red was added for staining for 15 min. The floating color was then washed away, followed by observation with a microscope.

Oil Red O staining: P3 BMSCs were seeded in a 24-well plate at a density of 1×10^5 cells/well. When the cell confluence reached about 60%, 1 mL of adipogenic inducer was added, and the medium was changed every three days for 21 days of culturing. After induction, the medium was aspirated and cells were washed with cold PBS three times. They were then fixed with 4% paraformaldehyde for 10 min and washed with water. Oil red O dye solution was added, dyeing 15 min in the dark, followed by a thorough wash with water to remove floating color. Then, observations and pictures were taken under a microscope.

Western Blot Analysis

Equal amounts of protein were mixed with an appropriate volume of 5× bromophenol blue loading buffer and boiled at 95 °C for 5 min. After gel electrophoresis and low temperature electric transfer, the PVDF membrane was incubated overnight with the corresponding antibody diluent (Tsg 101 rabbit polyclonal antibody (28283-1-AP, Protein Tech) was diluted at 1:2000; Rabbit Anti-CD63 polyclonal antibody (bs-1523R, Bioss) and Rabbit Anti-CD81 polyclonal antibody (bs-6934R, Bioss) were diluted at 1:1000; Dilution was prepared by adding 50 mL Tris7.6 (1 M), 8.775 g NaCl, and 1 mL Tween 20 to 1 L purified water.). Subsequently, the PVDF membrane was incubated with the secondary antibody dilution to analyze the blotting of the corresponding protein. The gray values of the protein bands were quantified through Image J.

Particle size and potential analysis

The purified TDN, Exo, and Exo-TDN were dispersed in PBS solution. Size distribution and particle concentration were determined through NTA using a NanoSight NS300. Zeta potential was determined through laser particle size analyzer using a BIC 9010.

Agarose electrophoresis

A total of 10 μL each from the synthesized DNA was taken, into which 2 μL 6× DNA loading buffer was added. The samples were mixed well and then loaded into the 3% agarose gel well, where they were run at 90 V for 30 min. A gel imager (QUANTUM, VILBER LOURMAT) was used to take pictures.

TDN, Exo-TDN and freshly extracted Exo were put into EP tubes. A DNA loading buffer was added and mixed in thoroughly, after which the samples were places into gel wells, where they were run at 90 V for 30 minutes. A gel imager was used to take pictures.

The cytotoxicity of DOX

The MTT method was used to evaluate the toxicity of DOX to cells. The final concentrations of doxorubicin were 0, 0.2, 0.4, 0.6, 0.8 and 1.0 μM. BRL-3A cells were seeded in a 96-well plate at a density of 1×10^3 to 2×10^3 cells/well, and incubated overnight for the cells to adhere. Then, the aforementioned different concentrations of DOX were added, followed by 24 h of incubation and then the addition of MTT. Absorbance was then detected with a microplate reader, and the survival rates of different groups of cells were calculated.

Material safety

The MTT method was used to evaluate the safety of the materials. In a 96-well plate, BRL-3A cells were seeded at a density of 2×10^3 cells/well. They were then incubated overnight to allow the cells to adhere. Then, different concentrations of TDN and exosomes were added, and after 24 h of incubation, MTT was added. Absorbance was detected by a microplate

reader, and the survival rates of different groups of cells were calculated.

Mouse primary hepatocytes were isolated by perfusion method. First, the perfusion solution was preheated in a water bath of 42 °C. Mice were anesthetized by isoflurane and fixed on foam plates. After sterilizing the mouse abdomen, open its abdominal cavity and locate the mouse hepatic portal vein. Gently peel off the mesangium around the portal vein with forceps and insert an indwelling needle at the free venous tube. Perfusion of Buffer I (The final concentration of each component was 0.5 mM EGTA, 25 mM HEPES, and 1% penicillin/streptomycin) flushed the blood in the liver and subsequently perfusion of buffer II (The final concentration of each component was 100 CDU/mL collagenase IV, 3 mM $CaCl_2$, 15 mM HEPES, and 1% penicillin/streptomycin) for digestion. After extracting the mouse liver tissue, split the liver envelope with forceps. Collect the digested hepatocytes and filter them using a 200 mesh screen. The cell suspensions were centrifuged at 500 rpm for 5 min, followed by collection of cell pellets. In a 96-well plate, primary hepatocytes were seeded at a density of 2×10^3 cells/well. Cells were used for further experiments after adherent cultures. Similarly, the protective effect of Exo-TDN was assessed according to the method mentioned above.

Cell uptake of Exo-TDN

The synthesized Exo-TDN (FAM labeled) was incubated with 20 mM of DiD at room temperature for 15 min, after which a 100 KDa ultrafiltration tube was used to centrifuge the sample at 4 °C and 4500× g to remove excess dye. This was then combined with the obtained DiD-labeled Exo-TDN and resuspended in PBS. Then, BRL-3A cells were inoculated in a 30 mm glass-bottom culture dish at a density of 1×10^4 cells/well. After the cells adhered to the glass, labeled Exo-TDN (20 μg/mL) was added for 4 h, 8 h and 12 h of incubation. The original medium was then aspirated and cells were washed with PBS three times, after which the nuclear dye Hoechst 33342 was added, followed by 10 min of incubation. Then, the samples were washed three times with PBS. The Exo-TDN uptake by the cells was then observed through a confocal fluorescence microscope or by flow cytometry.

The study of DOX uptake into the nuclei

The BRL-3A cells were inoculated in a 30 mm glass-bottom culture dish at a density of 1×10^4 cells/well. After the cells adhered to the well, 2 μM of DOX was added, followed by 24 h of incubation. The original medium was aspirated and cells were washed with PBS three times, after which the nuclear dye Hoechst 33342 was added. The samples were then incubated for 10 min and washed three times with PBS. The uptake of DOX by the cells was observed through a confocal fluorescence microscope.

The study of Exo-TDN polarized macrophages

RAW 264.7 cells were seeded in a confocal dish or six-well plate. After the cells adhered to the well, DiI (D8700, Solarbio)-labeled Exo-TDN was added for 4 h of incubation. The DiI-labeled Exo-TDN uptake by the RAW 264.7 cells was then observed through a confocal fluorescence microscope or by flow cytometry.

The experimental groups were the control group, the Exo-TDN group, the LPS+Exo-TDN group, and the LPS group. LPS solution was added to the LPS group and the LPS+PCM-Exo-TDN group (final concentration of 100 ng/mL). After 12 h, medium was replaced with fresh medium without LPS. Purified PCM-Exo-TDN was added to the Exo-TDN group and LPS+ Exo-TDN group (final concentration of 40 μg/mL). After 48 h of co-incubation, macrophage phenotypes were analyzed by flow cytometry and confocal fluorescence microscope. Among them, when the samples were observed by confocal fluorescence microscopy, three fields of view were selected for each well and the average of the fluorescence intensities in the three fields was calculated. Finally, the results of the three wells of each group are taken for statistical analysis.

The distribution of Exo-TDN in vivo

The synthesized Exo-TDN with 20 mM DiD was incubated at room temperature for 15 min and then put through a100KDa ultrafiltration tube at 4 °C. Centrifuging at 4500× g removed excess dye, after which the obtained DiD-labeled Exo-TDN was resuspended in PBS. When the tumors reached a volume of 80-100 mm^3, 20 μg of Exo-TDN (DiD labeled) was injected intravenously into the tumor-bearing mice. IVIS was then used to dynamically track the position of DiD-labeled Exo-TDN in the body, after which fluorescence images were captured at 4 and 24 h. After 4 h and 24 h, the mice were sacrificed. The hearts, livers, spleens, lungs, kidneys and tumors of mice were removed, and the enrichment of Exo-TDN in different organs was observed with IVIS.

Liver protection model

Tumor-bearing mice were divided into five groups, with six mice in each group. The experimental groups were the control group, the DOX group, the TDN+DOX group, the Exo+DOX group and the Exo-TDN+DOX group. The course of treatment was similar to that of the heart protection model. Treatment was for a total of 21 days.

Blood biochemistry and blood routine analysis

On the 21st day after treatment, blood was taken through the orbit, and approximately 1 mL of blood was collected from the orbit of each mouse for blood biochemistry and blood routine testing.

Statistical Analysis

GraphPad Prism 8.0 Software was used to perform the statistical analysis. All experiments were performed independently at least three times, and the results were presented as means ± standard deviation. Comparisons between any two groups were performed using the two-tailed, unpaired Student's t-test. Comparisons among more than two groups were performed using one-way ANOVA. Post-hoc testing was performed using the Turkey HSD method. Single, double, and triple asterisks represent $P < 0.05$, 0.01, and 0.001, respectively; $P < 0.05$ was considered statistically significant.

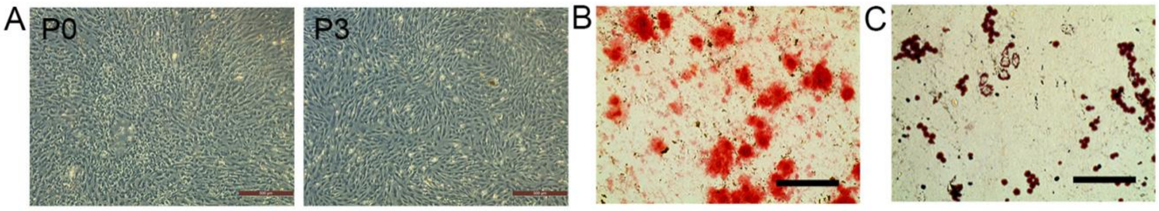

Figure S1. (A) Representative image of original (P0) and third (P3) generation BMSCs. Scale bars, 500 µm. (B) Study of the osteogenesis differentiation ability of P3 generation BMSCs. Scale bars, 200 µm. (C) Study of the adipogenic differentiation ability of P3 generation BMSCs. Scale bars, 200 µm.

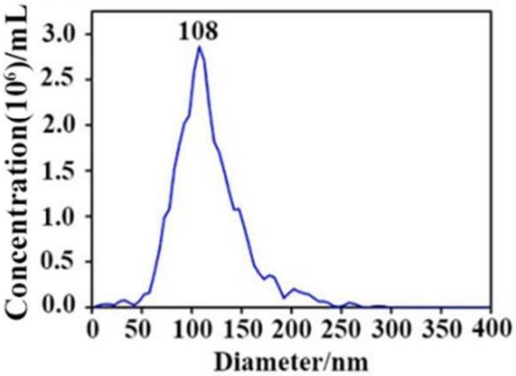

Figure S2. NTA result of the BMSCs-derived exosomes.

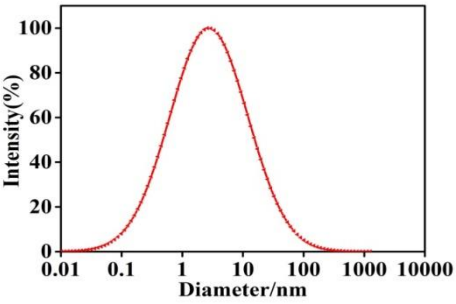

Figure S3. DLS result of TDN.

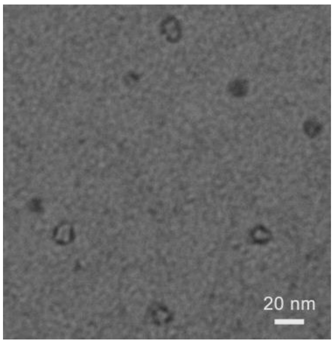

Figure S4. TEM result of TDN.

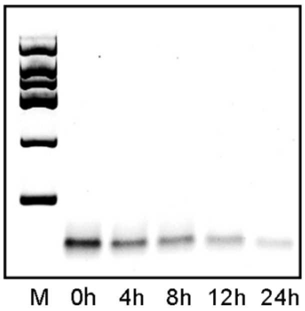

Figure S5. Agarose electrophoresis analysis of the stability of TDN incubation with 10% FBS for different time intervals.

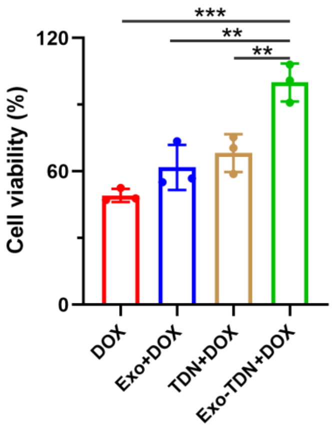

Figure S6. Cell survival rates of primary hepatocytes treated with DOX, Exo, TDN, and Exo-TDN (n=3).

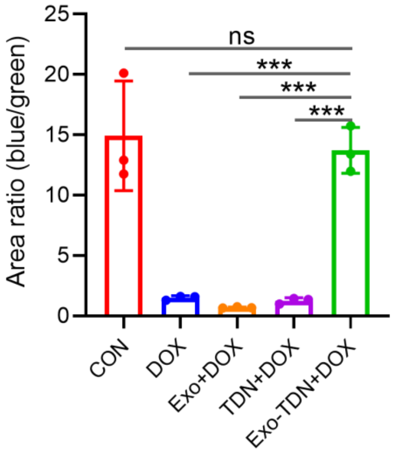

Figure S7. Quantitative analyses of LIVE-DEAD cell-staining images.

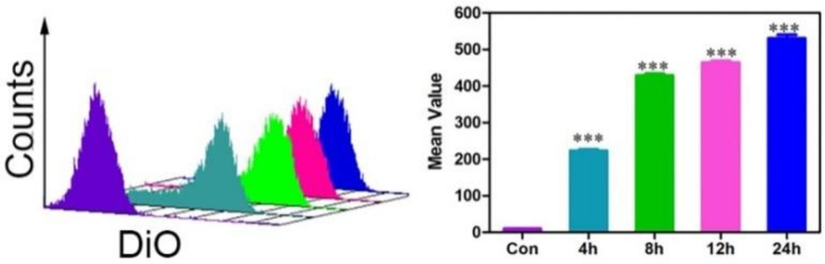

Figure S8. Flow cytometry results of BRL-3A cell uptake Exo-TDN.

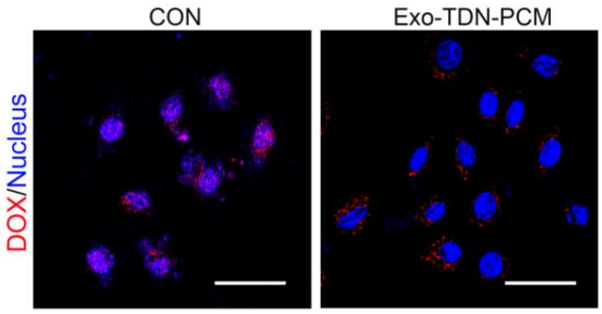

Figure S9. CLSM imaging of the inhibition of DOX from entering the nucleus of H9C2 cells by Exo-TDN-PCM. Scale bars, 50 μm.

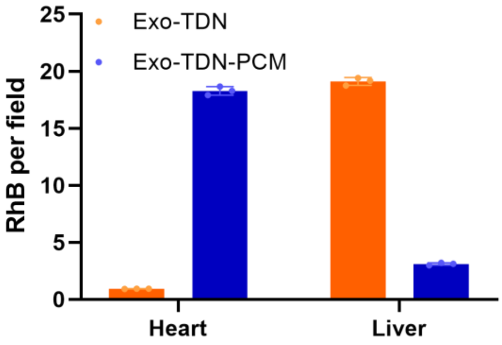

Figure S10. Fluorescence quantification results for Rhodamine B (RhB)-labeled Exo-TDN and Exo-TDN-PCM in the immunofluorescence images.

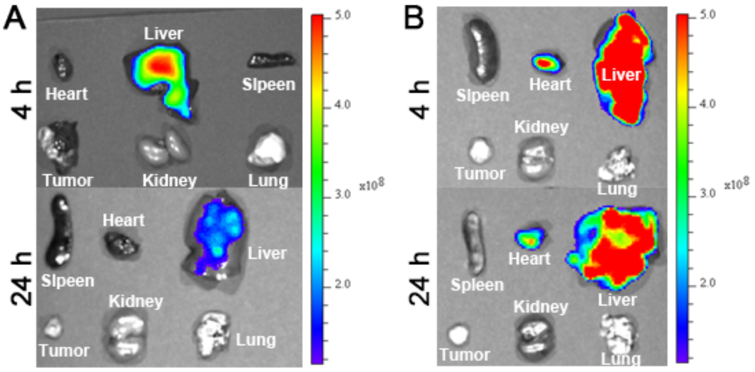

Figure S11. Ex vivo fluorescence imaging to detect the biodistribution of Exo-TDN (A) and Exo-TDN-PCM (B) after 4 h and 24 h of *i.v.* injection.

Table S1. Blood biochemistry results of mice in different treatment groups (n=4).

Groups	ALB	ALP	AST	ALT
Control	41.13 ± 8.07	47.48 ± 19.08	106.13 ± 24.17	68.35 ± 17.85
DOX	31.95 ± 14.35	270.08 ± 33.88	393.86 ± 43.46	495.10 ± 97.2
TDN+DOX	29.80 ± 14.5	294.13 ± 39.43	411.80 ± 42.7	487.68 ± 108.08
Exo+DOX	44.80 ± 20.8	184.53 ± 74.17	309.70 ± 30.4	361.32 ± 38.22
Exo-TDN+DOX	49.09 ± 16.51	70.57 ± 11.61	158.90 ± 38.3	149.56 ± 46.78

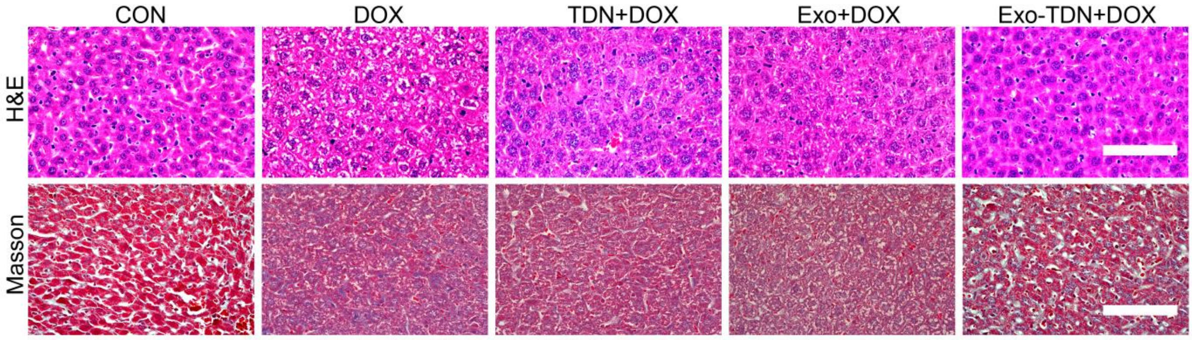

Figure S12. H&E staining and Masson staining images of liver tissue of mice in different treatment groups. Scale bars, 100 μm.

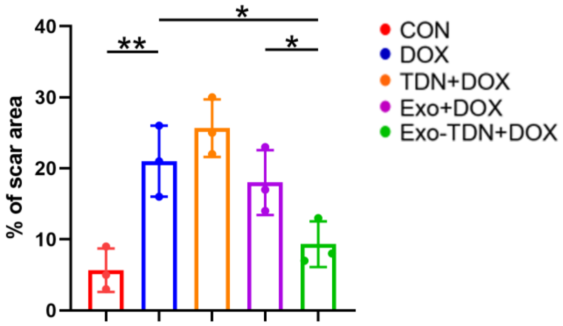

Figure S13. Quantitative analyses of Masson staining images of liver tissue (n=3).

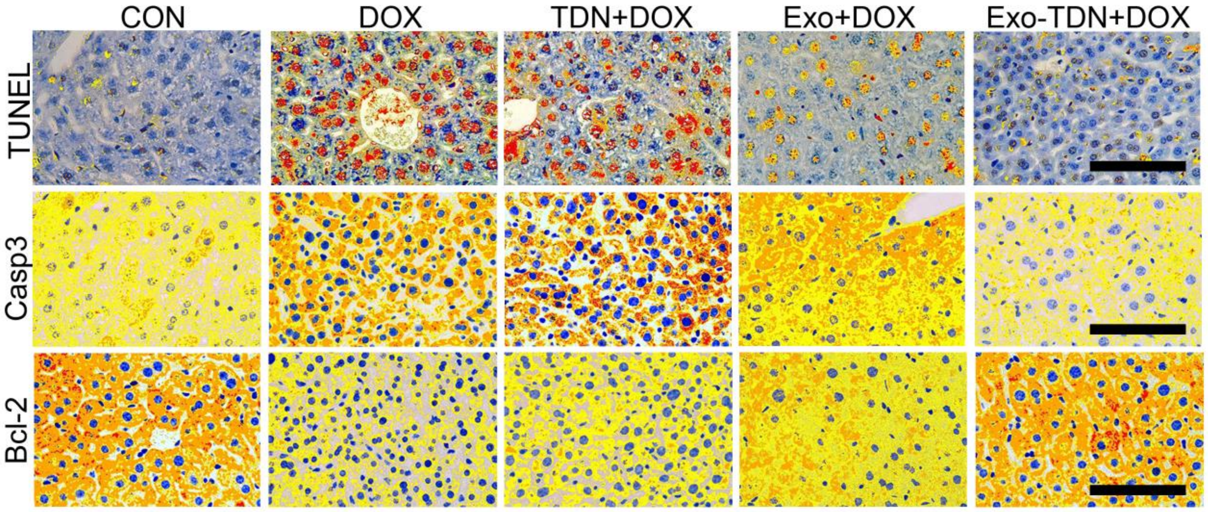

Figure S14. Histochemistry score images of TUNEL, Casp3 and Bcl-2 staining analysis of liver tissue in the treatment of different groups. Scale bars, 100 μm.

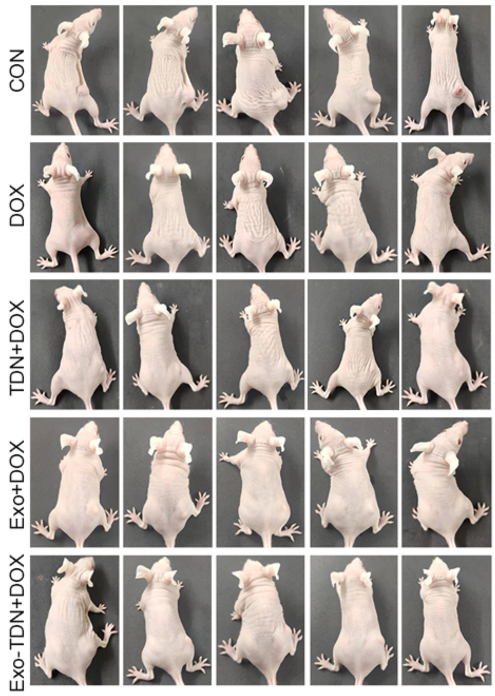

Figure S15. Representative photographs of tumor volume after 21 days of treatment.

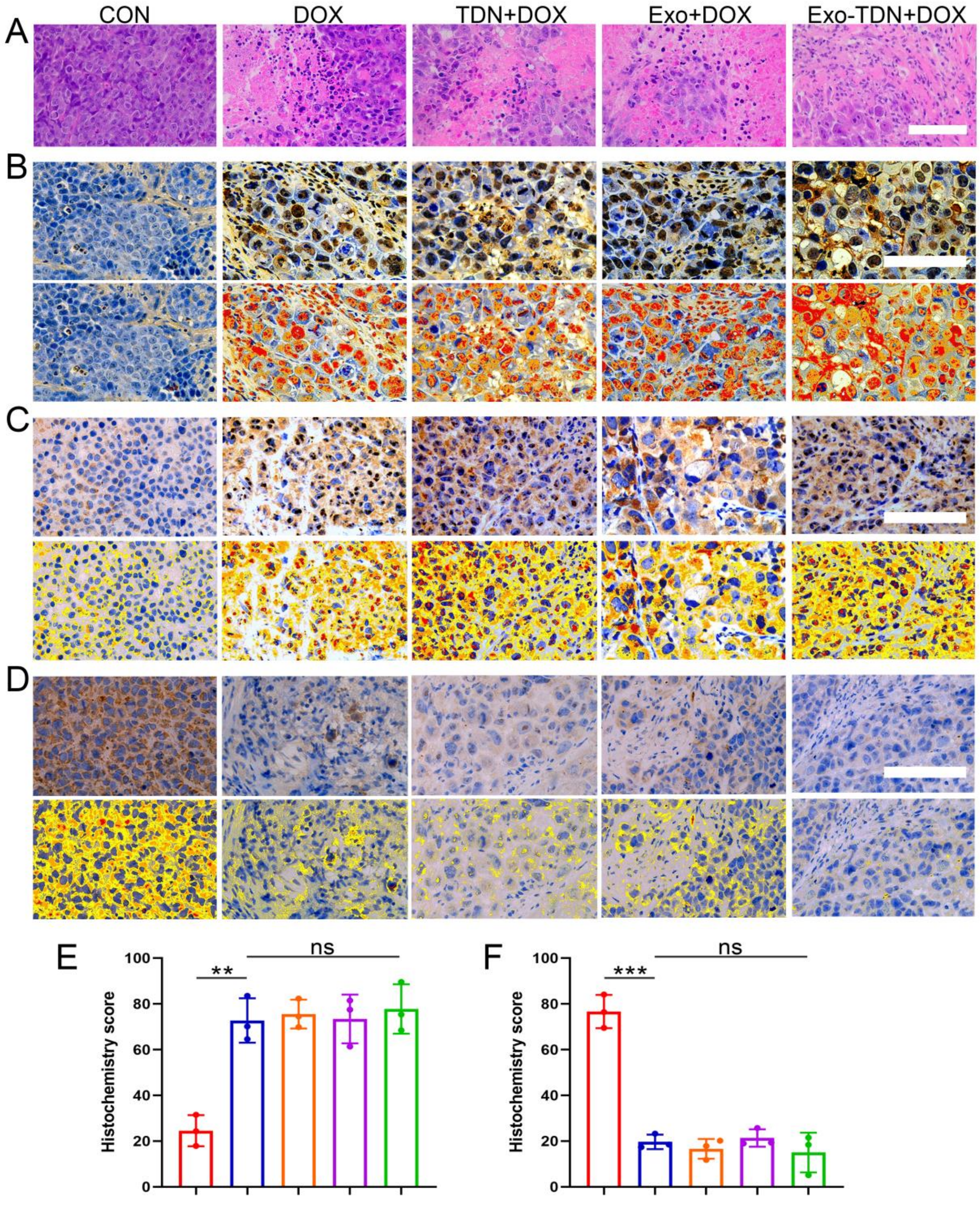

Figure S16. (A) H&E staining of tumor tissue in treatment groups. Scale bars, 100 μm. (B) TUNEL staining and histochemistry score images of tumor tissue in different treatment groups. Scale bars, 100 μm. (C) Immunohistochemical staining images and histochemistry score images of Casp3 in different treatment groups. Scale bars, 100 μm. (D) Immunohistochemical staining images and histochemistry score images of Bcl-2 in different treatment groups. Scale bars, 100 μm. (E-F) Histochemistry score of Casp3 (E) and Bcl-2 (F) staining images.

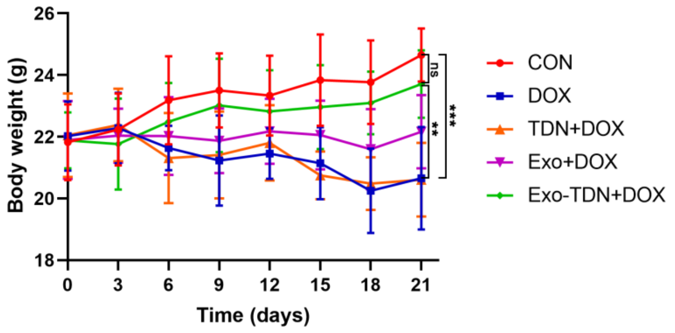

Figure S17. Body weight changes of mice in different treatment groups (n=6). Data are presented as the mean ± SD. *p < 0.05; **p < 0.01; ***p < 0.001; ****p < 0.0001.

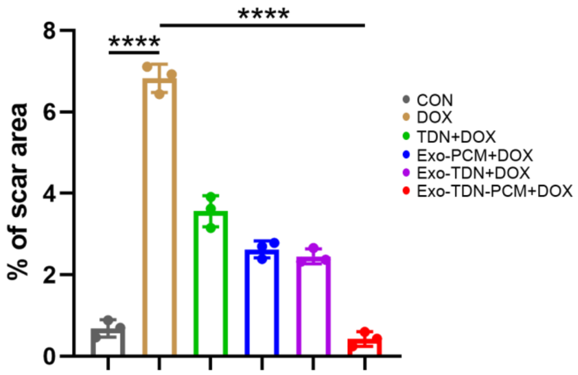

Figure S18. Quantitative analyses of Masson staining images of heart tissue (n=3).

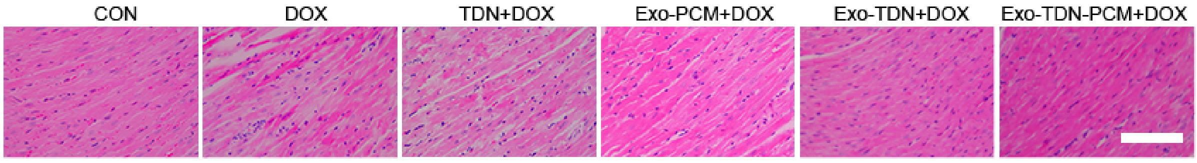

Figure S19. H&E staining images of heart tissue of mice in different treatment groups.

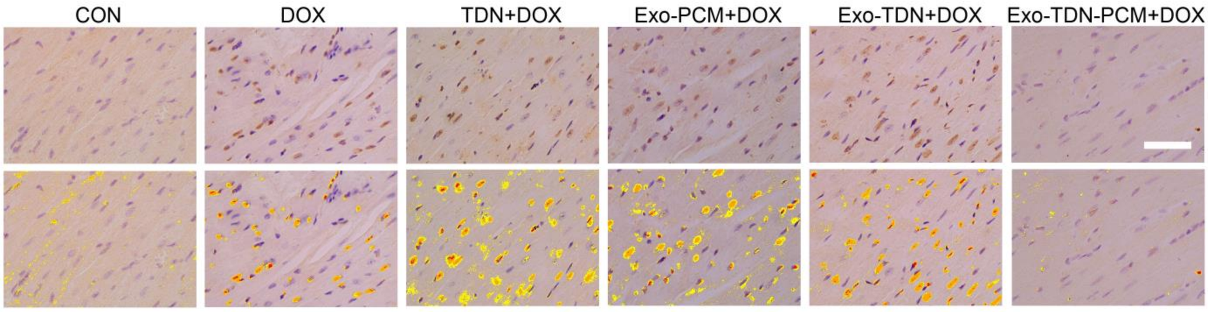

Figure S20. TUNEL staining images of heart tissue of mice in different treatment groups. Scale bars, 50 μm.

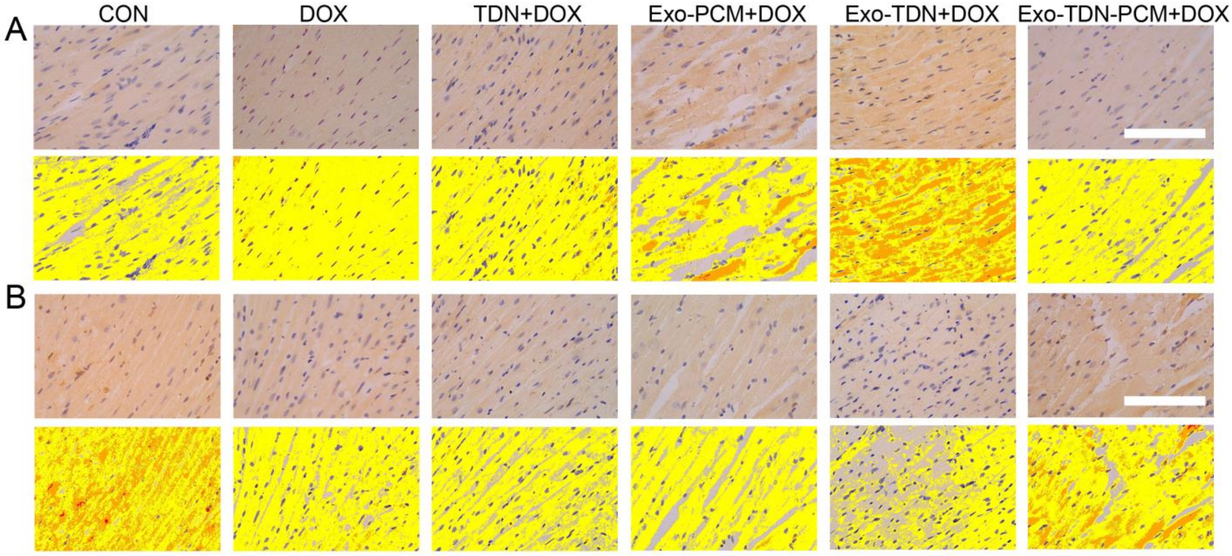

Figure S21. Immunohistochemical staining images of heart tissue showing Casp3 (A) and Bcl-2 (B) expression. Scale bars, 100 μm.

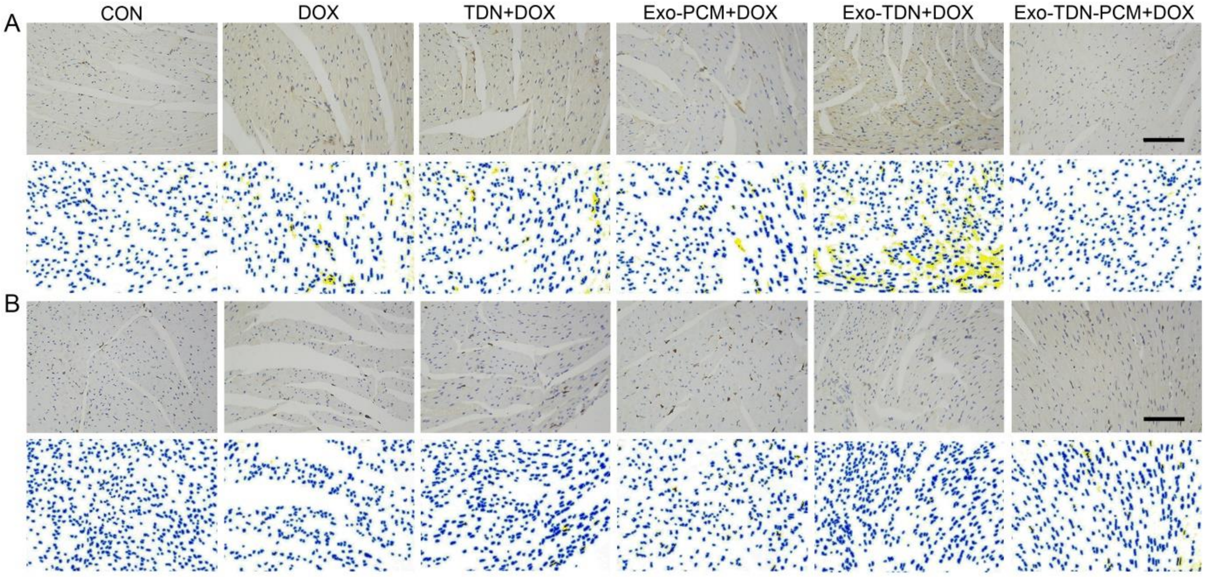

Figure S22. Immunohistochemical staining images and histochemistry score images of heart tissue showing CD80 (A) and CD206 (B) expression. Scale bars, 50 μm.

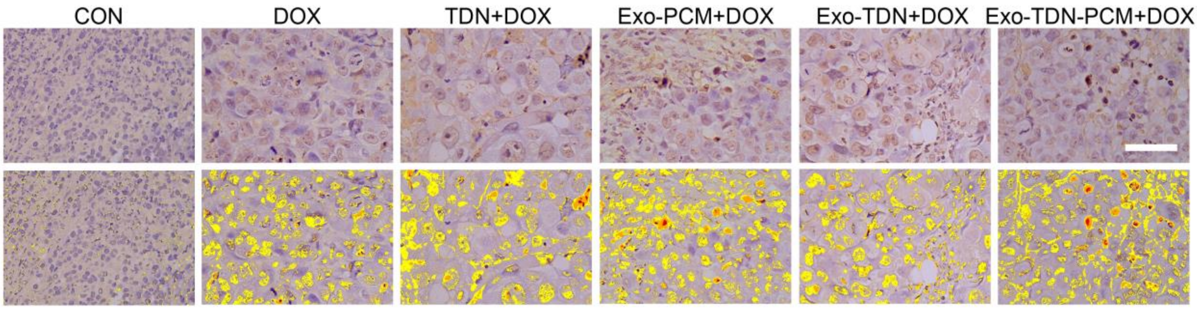

Figure S23. TUNEL staining images and histochemistry score images of tumor tissue of mice in different treatment groups. Scale bars, 50 μm.
